# An aquatic microrobot for microscale flow manipulation

**DOI:** 10.1038/s41598-022-07938-2

**Published:** 2022-03-23

**Authors:** Satishkumar Subendran, Chun-Fang Wang, Dineshkumar Loganathan, Yueh-Hsun Lu, Chia-Yuan Chen

**Affiliations:** 1 Department of Mechanical Engineering, National Cheng Kung University, Tainan, 701 Taiwan; 2 Department of Radiology, Shuang-Ho Hospital, Taipei Medical University, New Taipei City, 235 Taiwan; 3 Department of Radiology, School of Medicine, College of Medicine, Taipei Medical University, Taipei, 110 Taiwan; 4 Department of Radiology, National Yang-Ming University School of Medicine, Taipei, 112 Taiwan

**Keywords:** Engineering, Materials science

## Abstract

Microrobots have been developed and extensively employed for performing the variety tasks with various applications. However, the intricate fabrication and actuation processes employed for microrobots further restrict their multitudinous applicability as well as the controllability in high accuracy. As an alternative, in this work an aquatic microrobot was developed using a distinctive concept of the building block technique where the microrobot was built based on the block to block design. An in-house electromagnetic system as well as the control algorithm were developed to achieve the precise real-time dynamics of the microrobot for extensive applications. In addition, pivotal control parameters of the microrobot including the actuating waveforms together with the operational parameters were verified and discussed in conjunction with the magnetic intensity simulation. A mixing task was performed with high efficiency based on the trajectory planning and rotation control of the microrobot to demonstrate its capability in flow manipulation which can be advantageous for microreactor applications down the load. Aside from it, a dissolution test was further conducted to provide an on-demand flow agitation function of the microrobot for the next level of lab chip applications. The presented work with detail dynamic analysis is envisaged to provide a new look of microrobot control and functions from the engineering perspective with profoundly potential applications.

## Introduction

Microrobots are robots on a microscale that can accomplish high-precision operations in microenvironments. Such microscale operations are empowered by multimodal locomotion features^1^ including walking, jumping, rolling, and swimming driven or propelled by different external energy sources such as electric^2^, magnetic^3^, chemical^4^, acoustic^5^, and thermoelectromagnetic^6^ fields. Among all these, the utilization of a magnetic field for microrobot manipulation has unrivaled advantages, including remote maneuverability^7^, fuel-free propulsion^8^, reconfigurability, programmability^9^ and recyclability^10^ of magnetic materials, and versatility^11^ of combining with other external sources to achieve dual propulsion modes. Therefore, the affinity of different locomotion features activated by the magnetic field brings the substantial possibility to the applications of microrobot in various fields, including biology^12^, bioengineering^13^, medicine^14^, micromanufacturing^15^, and microfluidics^16–21^. In biomedical engineering, magnetically driven microrobots were employed for adaptive and multimodal locomotion^22^, carriers for therapeutic and targeted drug delivery^23^, acceleration for t-PA^24^ and for stem cell transplantation^25^. In the field of microfluidics, magnetically driven microrobots were employed to clean the surface contamination^26^, as force sensors for controlled locomotion^27^, and micromixers^28,29^. However, the control of the magnetic field distribution, precise target location, and a weak magnetic force that results in inadequate mixing are a few challenges incurred while endeavoring to scrupulously manipulate the magnetic microrobots in a confined microscale liquid environment.

Mixing at the microscale is an ongoing predicament. In microfluidic devices, mixing is limited by diffusion with low efficiency. Several researchers have been working on the design and development of micromixers for various applications. Based on the mixing principles, these micromixers are generally categorized as passive and active ones. In passive micromixers, the species are mixed due to specially designed channel geometry, and thus the external energy source is not required^30–34^. In contrast, external energy inputs such as acoustic^35^, magnetic^36^, electrical fields^37^, etc., are provided to generate advection in the active micromixers. Among them, the magnetic-field driven micromixers could enhance mixing efficiency by controlling ferrofluids, magnetic particles, and micro stirrers. Both permanent magnets and electromagnets have been applied to actuate micromixers. By simply displacing and rotating permanent magnets, it is possible to change the magnetic field intensity and direction easily. Zhou et al. found the formation of acceleration and deceleration couples nearer to the magnet channel due to the applied magnetic field. They pointed out that this mixing technique can be used to separate the target cell from the contaminated blood, protein synthesis, and processing enzyme in a microreactor^38^. Liosis et al. numerically studied the distribution of magnetic particles to separate and remove the heavy metal present in the contaminated water^39^. Saadat et al. achieved substantial mixing enhancement using the micro-magnetic coils embedded in the microchannel^40^. Apart from this, magnetically activated artificial cilia^16,28,36,41–47^ have been employed to regulate several hydrodynamic factors of the microfluidic network which results in significant flow manipulation. Though all these techniques appeared to enhance the mixing performance significantly, the strong dependence on the microchannel structure and a frail degree of controllability in flow manipulation alleviate the applicability of such devices to various microfluidic environments.

Although it requires electrical circuits to control electromagnets, it was suggested to generate the magnetic waveforms through electromagnets for profound applications where the representative dynamics of the micromixers for hydrodynamic advantages are necessary. Consequently, several studies have been conducted to achieve significant mixing by employing the electromagnetic system to propel the microrobot^48,49^. For example, Hyeonseok et al. achieved substantial enhancement in the mixing performance of safranin solution in deionized water^48^. Wang et al. designed an electromagnetically actuated microrobot (EAM) as a mobile micromixer that can be moved to the targeted position in a start-shaped microfluidic chip and controlled to produce rotary motion, which helps in achieving considerable mixing enhancement^49^. In the field of clinical medicine, the microrobots were designed as several small microrods^24,50^, microswarm^51^, and microwheel^52^ propelled by EMA that are employed to accelerate the t-PA by enhancing the convection induced by local vortex generation. Furthermore, to achieve localization mixing, several additional structures of high magnetic permeability, such as iron nails^24^ and magnetic spacer^53^, are added in the microfluidic channel which help locate the microrobot at a targeted position. Although the magnetic actuation microrobot has great potentials for medical applications, the adaptivity of microrobots from a laboratory setting to a clinical condition remains a daunting task.

The dissolution test is a globally necessitated test for most pharmaceutical products. Furthermore, this test is effectively acquainted in the industry as quality control to monitor the formulation^54^ and manufacturing process of the dosage by critically evaluating the drug bioavailability^55^ in the body. Recently in the field of medical engineering, several researchers have investigated the dissolution of doses combined with magnetic particles which are manipulated through the external magnetic field^56,57^. The dissolution rate of such magnetic-based drug composites is greatly influenced by the efficiency of magnetic manipulation. For instance, Gervasoni et al. experimentally studied the rate of sucrose powder dissolution, which was originally fabricated in a helical structure for magnetic manipulation. They reported that a rotating magnetic field of 30 mT resulted in the generation of corkscrew motion, which dissolves the entire sugar matrix in the deionized water in less than 20 minutes^56^. Similarly, iron^58^ or iron oxide^59^ and organic-based magnetic composites^60^ have been utilized to confer the chemical tunability without severely conciliating the magnetic moment. Periodically, the concept of dissolution has been employed in designing biodegradable microswimmers^61,62^. Such devices are propelled to deliver the drugs and degrade inside the human body by enhanced hydrodynamic interaction resulting in dissolution induced by magnetic manipulation, and hence these devices exhibit great importance to clinical applications^57^. Although the hydrodynamic interactions encountered by these techniques are still ill-defined, the reported significant shrinkage in such crystal structures has attracted more attentions for follow-up in-depth investigations.

In recent years several types of magnetic microrobots have been extensively investigated to demonstrate the intended tasks for various potential applications. Moreover, the shape of the magnetic microrobot can be tuned to promote the controllability of flow manipulation. For instance, Tottori et al. assembled the magnetic particles to produce a helical shape magnetic microrobot that controls the transportation and navigation direction induced by a rotating magnetic field^63^. However, assembling the magnetic nanoparticles to create 3D magnetic bodies such as helices, swarms, wheels, sperms, ribbons and other complex structures is still challenging which lessens the diversified applicability of such microrobots^57,64^. Therefore, there is an exigency to fabricate different architectural microrobots that inherently perform different tasks for various applications. One such striving conducive fabrication technique to abut this need is by channeling the magnetic building blocks for magnetic microrobots. Besides deftness in fabrication, the complete exposure of such architecture to the external magnetic field enhances the propulsion efficiency, resulting in precise real-time spatial localization. Therefore, in this pilot work, the aquatic microrobot was fabricated by employing the magnetic building block technique where the microrobot was built on the block to block design. Furthermore, this study was focused on investigating the control parameters of the microrobot, including the actuation waveforms and the operational parameters, and these parameters were further verified and discussed in association with the magnetic intensity simulation. To demonstrate the capability of the microrobot in achieving high-efficiency flow manipulation, the trajectory planning and the rotation control of the microrobot were investigated through mixing and dissolution tasks. The associated in-depth investigations and pertain discussion were documented and provided in this work as proof of demonstration and future applications.

## Methods

### Microrobot fabrication

**Figure 1:**
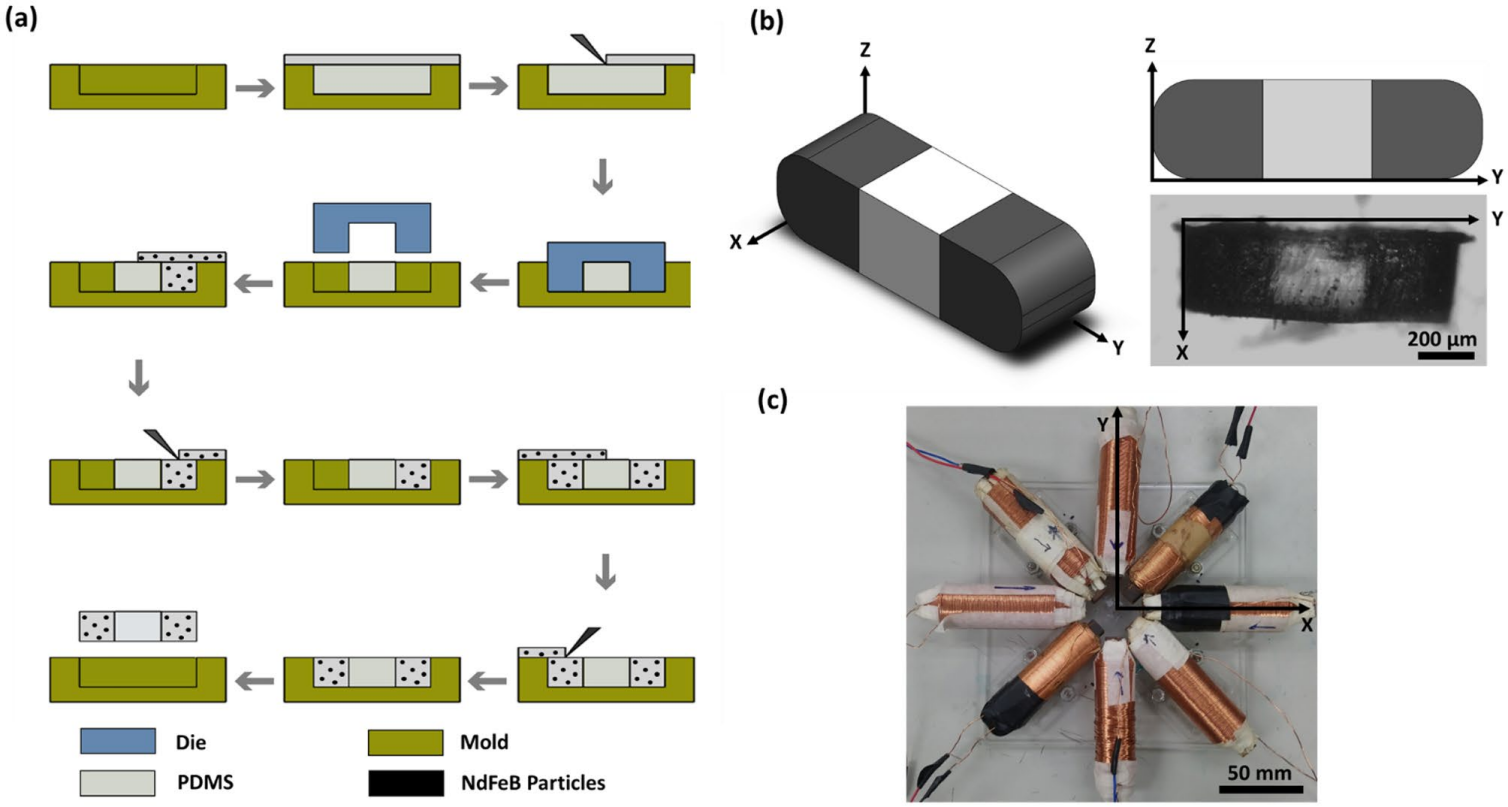
Fabrication of the aquatic microrobot and electromagnetic actuation system. (**a**) The fabrication process of the microrobot. (**b**) Geometry of the microrobot, top-left is the isometric view of microrobot design; top-right is the top front view of microrobot design; the bottom-right is the top view of the microrobot under microscope imaging. (length: 1000 µm, width: 300 µm, Height: 300 µm). (**c**) Setup of the in-house eight-coil electromagnet platform for the microrobot control.

The layout of the microrobot consisted of three parts, one of which was rendered by polydimethylsiloxane (PDMS, Sylgard 184, Dow Corning Corp., Midland, MI, USA) and the other two by a mixture of NdFeB magnetic particles (MQP-15-7, Magnequench, Singapore) and PDMS. Meticulously, a mixing proportion of 4:1 was chosen by considering the trade-off of sensitivity and mechanical property of the microrobot. In order to preclude this mixture from bubbles formation, the mixing process was eventuated in a vacuum environment for over 20 min. Additionally, the mixture was magnetized to procure a homogenous distribution of the particles, thereby preventing agglomeration. Upon successful magnetization, the mixture was turned into a thixotropic paste, a time-dependent shear thinning state. As shown in the Fig. 1a, a molding method was implemented to fabricate the segmented structures, and with the help of acrylic blockers, the mixture was poured into the mold and cured sequentially. The mold and block made of acrylic was manufactured using CNC machining tool. To balance the magnetic force acting on the microrobot and decrease its oscillation, the two magnetic segments were magnetized with distinct magnetic field directions. The design and geometry of the microrobot is illustrated in Fig. 1b respectively.

### The magnetic coil system

An in-house eight-coil magnetic system was designed and built as illustrated in Fig. 1c by adopting the basic structure of the previous research ^12,44,46,65^. The electromagnet was built by wrapping the enameled wire around a steel bar with a total of 1200 turns. The intensity of the electromagnet was evaluated. With measured 0.3 A of the peak current in single coil, the peak intensity of one coil was around 500 mT.

**Figure 2:**
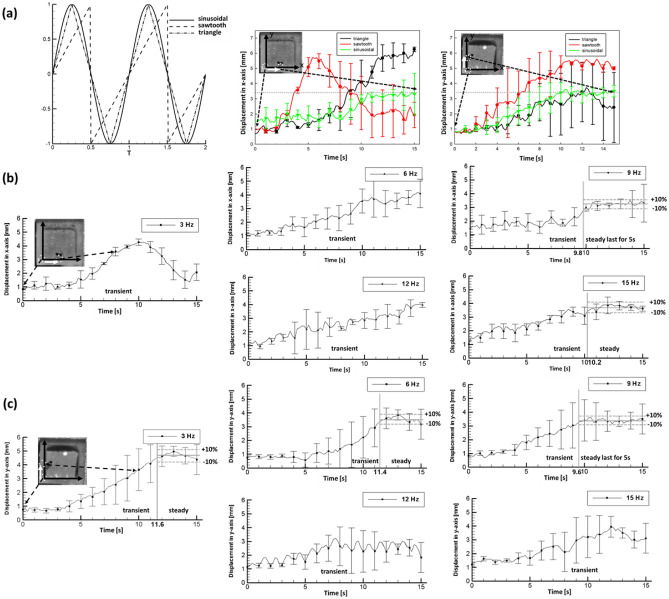
Performance of the microrobot under different control parameters. (**a**) Typical illustration of all the three waveforms, moving trajectories in x and y-direction of microrobot controlled by triangle (black curve), sawtooth (red curve), and sinusoidal (green curve) waveforms with 9 Hz of frequency respectively along with the microphotographs of the microrobot. (**b**) Moving trajectories in x-direction and (**c**) y-direction of the microrobot controlled by sinusoidal waveform with 3, 6, 9, 12, and 15 Hz of frequency. Error bars represent one standard deviation in either direction from three repeated trials.

A data acquisition device (NI cDAQ-9174, National Instruments, Austin, TX) with modules embedded for signal inputs and outputs (NI 9201 and 9264) was connected to the coils and an external power supply to facilitate the motion control of the microrobot. LabVIEW (National Instruments), a graphical programming language, was adopted to create a manipulation interface that could simultaneously modify the control parameters of the microrobot together with the operational parameters to undergo dynamic motion. The magnetic field of both constant and varying magnitude that varies in direction was generated using the proposed electromagnetic system. To magnetically steer the microrobot in the direction of interest, the magnetic force and magnetic torque were induced in the microrobot, which resulted in translational and rotational motions. Additionally, the magnetic intensity simulation was carried out to support the experimental data on the magnetic field, including the magnetic flux density and gradient. A detailed dynamics of translational and rotational motions together with numerical validation are provided in the supplementary material. Further, the operational parameters were examined under three different waveforms to facilitate smooth trajectory planning and robust rotational control of the microrobot. The performance of the microrobot under three different types of waveforms, as illustrated in Fig. 2a, was examined. The three types of waves showed different characteristics: a sudden drop occurred in each cycle of the sawtooth wave, a triangle waveform was composed of two linear lines, and a sinusoidal wave showed the smooth curve. Those waveforms can be used to modify intensity of each electromagnet, from a sharp drop to a smooth transition.

### Imaging and analysis

A charge coupled device (CCD) camera (WAT-902H ULTIMATE, WATEC, Japan) connected with a micro lens (AF Micro-NIKKOR 60 mm f/2.8D, Nikon, Japan) was used for recording, with 30 of the frame rate, and 250 × 250 of resolution for the clips in supplementary movies. The ability of the microrobot to mix chemicals in the blood was evaluated by Eq. (1) through the intensity of pixels (m_i_), average intensity of an image to measure the fluid uniformity ($\overline{m }$) and (n) represents the total number of pixels in an image. The acquisitions of information of images including pixel numbers and intensity were achieved with image processing software, ImageJ.

1 $$\mathrm{Mixing\,effeciency }\left(\mathrm{\%}\right)=(1-\frac{1}{\overline{m} }\sqrt{\frac{\sum_{i}^{n}({{m}_{i}-\overline{m })}^{2}}{n}})\times 100.$$

### Shrinkage percentage

Shrinkage was considered as the reference to evaluate the efficacy of microrobots in the dissolution experiments. The levels of shrinkage were measured by Eq. (2), in which the shrinkage percentage was the ratio of instant area and initial area of a crystal, ${n}_{t}$ and ${n}_{0}$ representing the number of pixels that a crystal took at time = t s and time = 0 s, respectively.

2 $$\mathrm{Shrinkage\,percentage}=\left(1-\frac{{n}_{t}}{{n}_{0}}\right)\times 100.$$

## Results

### Microrobot dynamics

The performance of microrobot under three different types of the waveforms, namely, sinusoidal, sawtooth, and triangle, are illustrated in Fig. 2a. Several trials were performed by varying frequency ranges (3, 6, 9, 12, and 15 Hz) under the applications of three different waveforms using LabVIEW. Fig. 2a, illustrates the displacement curves of the microrobot in the x and y-direction under the applications of the different waveforms. Especially, for the case of the triangle and sawtooth waveforms, the microrobot trajectory path over the time period was in a transient state. As, illustrated in Fig. 2a the displacement of the microrobot in the x and y-direction for triangle and sawtooth waveforms showed that the microrobot path was not consistent even after 10 s whereas for the sinusoidal waveform the microrobot achieved dynamic motion with small oscillation after 10 s in both the directions. Certain observations such as overshoot, drawback, and large standard deviation were evaluated when the microrobot was operated using triangle and sawtooth waveforms. These findings determined the unstable state of the microrobot. In addition, amplitude of oscillation of average value was considered in the assessment of the performance of the microrobot in x and y-direction. As illustrated in Fig. 2b for 3 Hz and 6 Hz the displacement of the microrobot in x-direction significantly dropped and increased over the selected time period. In addition, the microrobot achieved dynamic motion with small oscillation after 11.5 s while moving in the y-direction (See Fig. 2c). Similarly, for 12 Hz the displacement of the microrobot in both the directions was not consistent. In 15 Hz, the microrobot achieved dynamic motion with small oscillations after 10.2 s while moving in the x-direction whereas it was in transient in the y-direction (see Fig. 2c).

It was concluded that the microrobot had better performance (in terms of small oscillation) under 9 Hz sinusoidal signal as compared to the triangle and sawtooth waveforms. Thus, further investigation towards the trajectory path of the microrobot using sinusoidal waveform under different frequency was considered (See Supplementary Video 1). In Fig. 2b,c, a rotating magnetic field was applied to control the motion of the microrobot in deionized (DI) water (See Supplementary Video 1). The sinusoidal signal-actuated microrobot could travel to the center (3.5 mm) of the edges of the tank whereas the other two forms of signal-actuated microrobot would overshoot or withdraw. Figure 2b,c illustrate the sinusoidal signal output of the microrobot at different frequencies (3, 6, 9, 12, and 15 Hz). It was observed that the microrobot was in transient state for about 15 s in both the x and y-directions, under the frequency range (3, 6, and 12 Hz). Particularly for 15 Hz, the microrobot achieved dynamic motion with small oscillation for about 5 s in the x-direction whereas the microrobot was in transient state while displacing along the y-direction. In addition, the performance of the microrobot under sinusoidal waveform was the most stable with a mean standard deviation (SD) of 0.54 mm whereas for triangle and sawtooth waveforms the deviations were larger (1.15 mm and 0.81 mm). The dynamic motion with small oscillation of the microrobot was achieved as result of the edge to edge translating rotational motion. This was achieved by precise control through the continuous actuation of the microrobot towards the edges of the tank. In addition, the microrobot travels along this path by rotating itself thus generating a chaotic flow in the environment. As illustrated in the Fig. 2b,c, the fastest dynamic motion with small oscillation achieved by the microrobot was achieved within 10 s. With a frequency of only 9 Hz, the microrobot was able to travel to the target location and stay stable for more than 5 s in both the x and y-directions whereas in other cases the microrobot took comparatively longer fraction of seconds to reach the dynamic motion with small oscillation.

### Mixing performance

**Figure 3:**
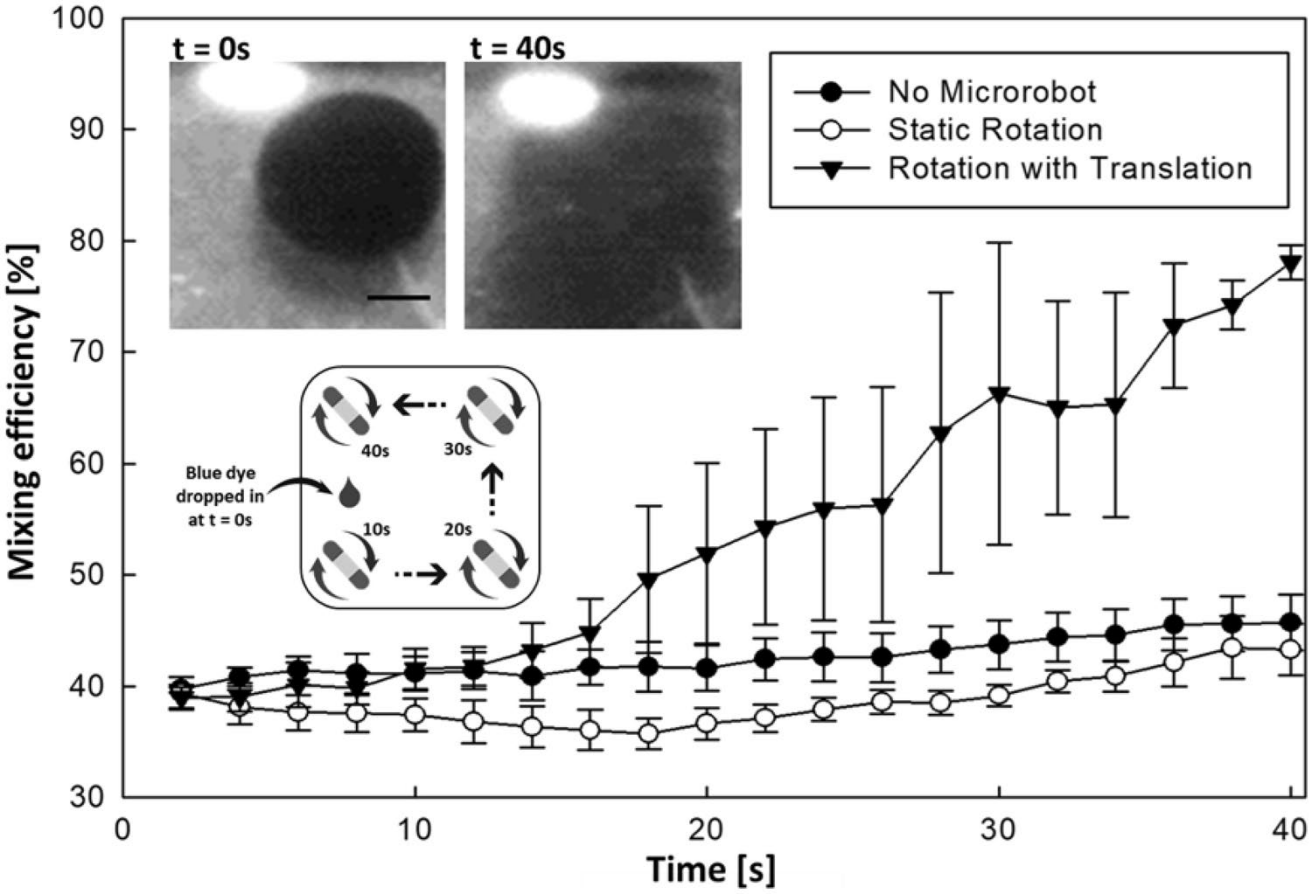
Effects of the magnetic actuation on the mixing efficiency in the absence of microrobot (mode I), under static rotation (mode II), and rotation with translation (mode III). In the top-left, Microphotographs (time-lapse snapshots) captured during moving speed edge to edge moving speed for mixing enhancement at t = 0 s and t = 40 s. In the bottom-left, Schematic illustration of the microrobot’s motion (edge-to-edge rotation with translation motion) in water under a rotating magnetic field for various time period (t = 10, 20, 30, and 40 s). Error bars represent one standard deviation in either direction from three repeated trials. Scale bar is 1 mm.

The mixing performance of the microrobot was evaluated based on three different modes as illustrated in Fig. 3. Initially at t = 0 s, a distinct flow boundary of blue dye in water (static) can be observed clearly. To perform mixing, the microrobot was actuated (using mode III). This resulted in deformation of the fluid streamline due to the hydrodynamic drag force. Thus, effectively raising the contact area between the dye and the water. To accelerate mixing, the microrobot was moved within the edges of the tank and rotated clockwise at a frequency of 9 Hz. It was noted that the mixing efficiency drastically boosted from 40 to 80% in 40 s (see Fig. 3). This is due to the edge-to-edge moving strategy of the microrobot boosted the mixing efficiency over the time-period significantly (See Supplementary Video 2). During the first half (until 10 s) there was no significant changes observed in all the three modes (See Fig. 3). Whereas, in the second half (10 s to 40 s) there was a substantial increase in the mixing performance observed in mode III (rotation with translation). Differences between the mixing performances of all the three modes were quantified by calculating the slope values during the second half. The results showed that the time required for mode III (rotation with translation) to reach the highest mixing performance was 5.18 times faster than that for mode II (static rotation). In addition, the highest mixing performance in mode III was evaluated as 80%, whereas that in mode II and mode I was between 39 to 42% respectively. It thus inferred that the microrobot actuation with translation rotation from edge to edge could boost the mixing efficiency of the local region.

### Dissolution of NaCl

**Figure 4:**
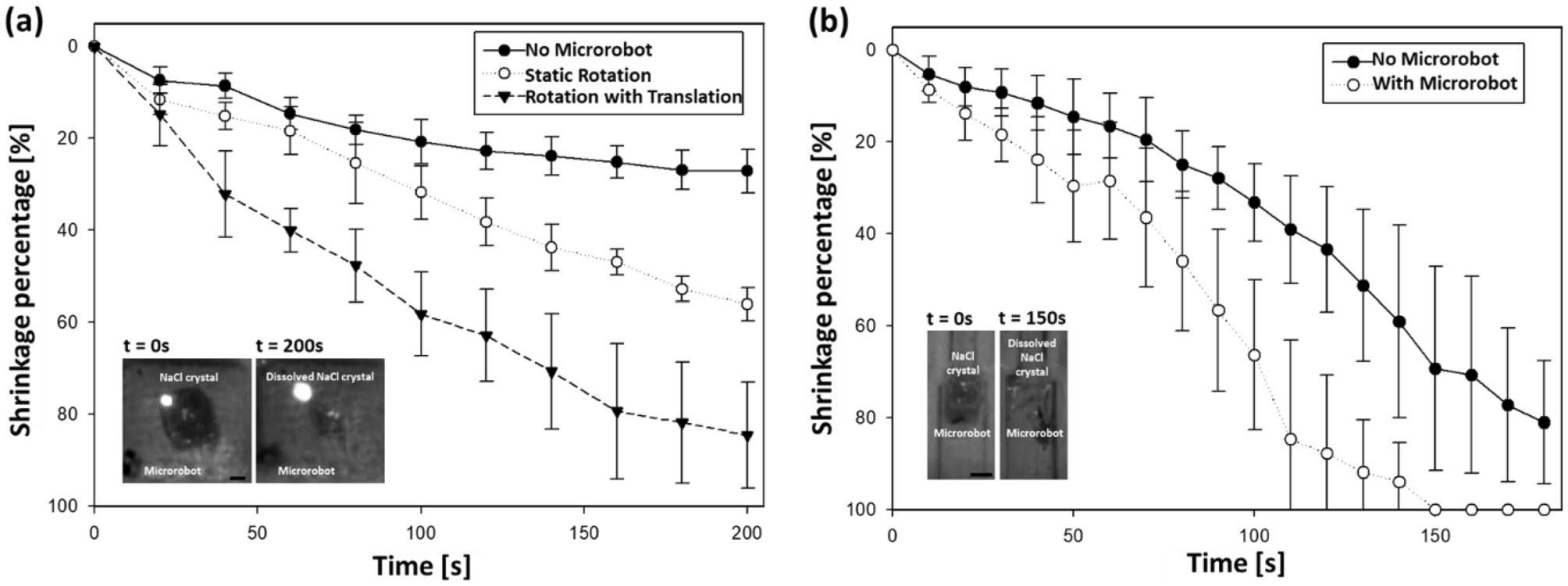
Performance of the microrobot in dissolution of NaCl crystal. (**a**) Shrinkage percentage of NaCl crystal dissolved in an open tank in the absence of microrobot (mode I), under static rotation (mode II), and rotation with translation mode (mode III). Microphotographs (time-lapse snapshots) captured during rotation with translation mode at t = 0 s and t = 200 s. (**b**) Shrinkage percentage of NaCl crystal dissolved in the closed channel in the absence of microrobot, and with microrobot. Microphotographs (time-lapse snapshots) captured during the second mode (with microrobot) at t = 0 s and t = 150 s. Error bars represent one standard deviation in either direction from three repeated trials. Scale bar is 1 mm.

As a part of further investigation, dissolution experiment was facilitated using the fabricated aquatic microrobot. The dissolution experiment was performed in two conditions—in an open tank and in a closed channel. The closed channel was designed with similar scale to cerebral arteries, with 2 mm of width. Also, the flow rate of the closed channel experiment was set to 80 ml/min, similar to the flow occurring in anterior cerebral artery (ACA)^66,67^. The challenge of the microrobot was to dissolute the NaCl crystal in both the conditions. Figure 4a illustrates the dissolution shrinkage percentage performed in an open tank under three modes. The microrobot was autonomously driven from the starting point to the target location (NaCl crystal) using the EMA system. It was observed that shrinkage percentage was relatively low in the mode I (in the absence of microrobot) and mode II (static rotation). The average shrinkage rate (at t = 200 s) for both the conditions were 0.136% and 0.280%, respectively. Contradict to this, the average shrinkage rate in mode III (rotation with translation) was 0.422%, which was significantly higher as compared to mode I and II. As illustrated in Fig. 4b for the closed channel, the average shrinkage rate (at t = 150 s) was 0.450% without microrobot, while the shrinkage rate was 0.667% with microrobot. The SDs showed an increasing trend with the increase in shrinkage percentage which was due to the unstable conditions in the fluid environment as the NaCl crystals dissolute and get displaced in the surrounding environment. During the dissolution, the NaCl crystals would initially shrink from the four edges of the tank and form star-liked shape, over the time the crystals breakdown into irregular shapes while reaching the end of the process (See Supplementary Video 3 and 4). As illustrated in the microphotograph in Fig. 4a at t = 200 s during mode III the microrobot dissolute the NaCl crystal with shrinkage of 80% respectively.

## Discussion

For achieving dynamic motion with small oscillation and precision of the microrobot within the fluidic environment, adequate control parameters were identified. For the initial investigation, the microrobot was placed in an acrylic tank (7 x 7 mm), the whole setup was arranged in the center of the EMA system. The strength of the magnets was balanced to navigate the microrobot from the corner to the center of the two edges, attempting to hold it in the center by tracking the trajectory path of the microrobot. Different waveforms and rotation frequency of output signals from DAQ, which determined the intensity of electromagnets, were considered. The experimental results validate the ability of the aquatic microrobot to translate and keep small oscillation within the environment. It was observed that the performance of microrobot under sinusoidal waveform at a frequency of 9 Hz resulted in reaching the target position at earliest and achieved dynamic motion with small oscillation for about 10 s, possessing a small amplitude of oscillation (< 0.3 mm).

As the proof of concept, the mixing performance of the microrobot was evaluated under different conditions (See Fig. 3). It was observed that the mixing percentage was significantly enhanced from 40% to 80% in 40 s. The experiment results highlighted that rotation with translation motion is suitable to improve the mixing efficiency in a confined environment. Further to demonstrate micro-manipulation tasks using the aquatic microrobot, a dissolution experiment was performed. The microrobot was actuated in a controllable manner to dissolve the NaCl crystal (target). It was observed that microrobot actuated under mode III (open channel) significantly dissolved the NaCl crystal (See Fig. 4a). Similarly, the microrobot was actuated within the closed channel was used to evaluate the shrinkage percentage. It was observed that the time required to remove the blockage (NaCl crystal) in the channel was shortened by about 30 s by the microrobot (See Fig. 4b). These results show that the aquatic microrobot is capable of dissolving NaCl crystal with the potential for *in-vitro* biomedical applications. In order to further demonstrate the capability for the dissolution of biological depositions in an appropriate condition, this study may be expanded to remove the blood clot by using the fabricated microrobot. As deleterious biological aggregation processes, such as blood clotting for thrombi, directly cause many medical complications and is a major threat to our health. The controllable disruption and subsequent removal of blood clots are of great importance for the treatment.

To summarize, we have introduced the distinctive fabrication technique in which the microrobot was built based on the block-to-block design. Compared to the existing fabrication methods, the concept of the building block technique has several advantages, such as (1) it provides the simple and facile way to assemble the magnetically active components in creating three-dimensional microstructures. It enables efficient production of microrobot architectures in different shapes including H, T, X, etc., in which the magnetically active components can be arranged at the corners of these configurations for better dynamic motions. (2) The proposed electromagnetic system, together with the developed control algorithm, can be employed to direct the microrobot in a variety of reconfigurable locomotion styles. This feature facilitates the microrobot to propel along the desired trajectory and hence signifies the attainability of real-time dynamics applicable to various microfluidic environments for multitasking operations. Such property of aquatic microrobot is essentially important for enabling better hydrodynamic control, especially in the field of cardiovascular surgeries.

## Supplementary Information

Supplementary Video 1.

Supplementary Video 2.

Supplementary Video 3.

Supplementary Video 4.

Supplementary Information 1.

## Data Availability

All data are available in the main text or the supplementary materials.
