# Supplementary material for: An aquatic microrobot for microscale flow manipulation
Source: Sci Rep. 2022 Mar 23;12:5041. doi: 10.1038/s41598-022-07938-2 (PMC8942993; doi:10.1038/s41598-022-07938-2)
Supplement: Supplementary file 5 — Supplementary Information 1. [file 41598_2022_7938_MOESM5_ESM.docx]

**Electronic Supplementary Information (ESI) for**

**An aquatic microrobot for microscale flow manipulation**

By

**Satishkumar Subendran^1,^ ^†^, Chun-Fang Wang^1,^ ^†^, Dineshkumar Loganathan^1,^ ^†^, Yueh-Hsun Lu^2, 3, 4^, and Chia-Yuan Chen^1, *^**

†Equal contribution

^1^Department of Mechanical Engineering, National Cheng Kung University, Tainan 701, Taiwan ^2^Department of Radiology, Shuang-Ho Hospital, Taipei Medical University, New Taipei City 235, Taiwan

^3^Department of Radiology, School of Medicine, College of Medicine, Taipei Medical University, Taipei 110, Taiwan

^4^Department of Radiology, National Yang-Ming University School of Medicine, Taipei 112, Taiwan

*****Correspondence: chiayuac@mail.ncku.edu.tw; Tel.: +886-2757575-62169

**Introduction**

This supplementary document is divided into two sections for detailed discussions.

Section 1: The control strategy for microrobot locomotion.

Section 2: A theoretical analysis on microrobot dynamics.

Supplementary Video 1: Motions of the microrobot under sinusoidal waveform at 9 Hz.

Supplementary Video 2: Micromixing performance of the microrobot.

Supplementary Video 3: Dissolution of NaCl in an open channel.

Supplementary Video 4: Dissolution of NaCl in a closed channel.

**1. The control strategy for microrobot locomotion**

- 1. **Experimental setup**

A controlled extracorporeal control system was designed and employed to control the dynamics of the microrobot. An external magnetic source consisting eight electromagnetic coils was placed spanning circumferentially to the microrobot as shown in the Fig. S1. This system provided the control of the translational and rotational movements of the microrobot.

| (a) | **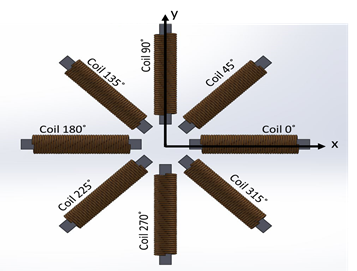** | (b) | **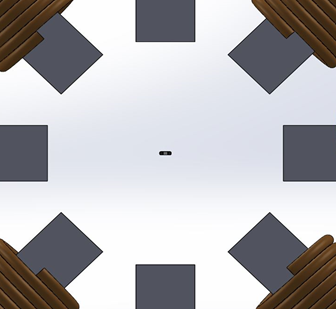** |
| --- | --- | --- | --- |
| **Fig. S1** An external magnetic source setup. (a) The coordinate and the arrangement of the electromagnet coils. (b) A zoom-in image exhibits the initial position of the microrobot with respect to the coil system. | | | |

**1.2 Rotational and translational motions of the microrobot**

As known that the magnetic torque and forces acting on the microrobot resulted in the microrobot to displace and rotate to the desired locations. Before the discussion on the translational and rotational mechanisms of the microrobot, it is necessary to describe how the microrobot was balanced in a static equilibrium condition under the influence of a magnetic field.

Assumptions made based on the position of microrobot and coil:

(1) Throughout the experiment, the initial position of the microrobot was set at the center of the platform which is at the midpoint between two coils facing each other, as shown in Fig. S1(b).

(2) The eight coils are identical, and that generated a magnetic field pointing away from the origin while applying the positive current on them.

(3) Only the interaction of the microrobot with coils at 0-degree and 180-degree is considered to provide basic concept of the microrobot control.

**1.2.1 Static equilibrium conditions of the microrobot**

Figure. S2(a) shows an antisymmetric magnetic field distribution over the microrobot when a current of the same magnitude was applied on the coils kept at 0˚ and 180˚. The magnetic field gradient around the origin is shown in Fig. S2(b), and it was found that the gradient distribution was symmetric. The magnetic forces acting on the two magnetic sections of the microrobot, F_B,1_ and F_B,2_ were of the same magnitude and opposite in directions as shown in Fig. S2(c) and with such arrangement the microrobot was kept in a stable fashion.


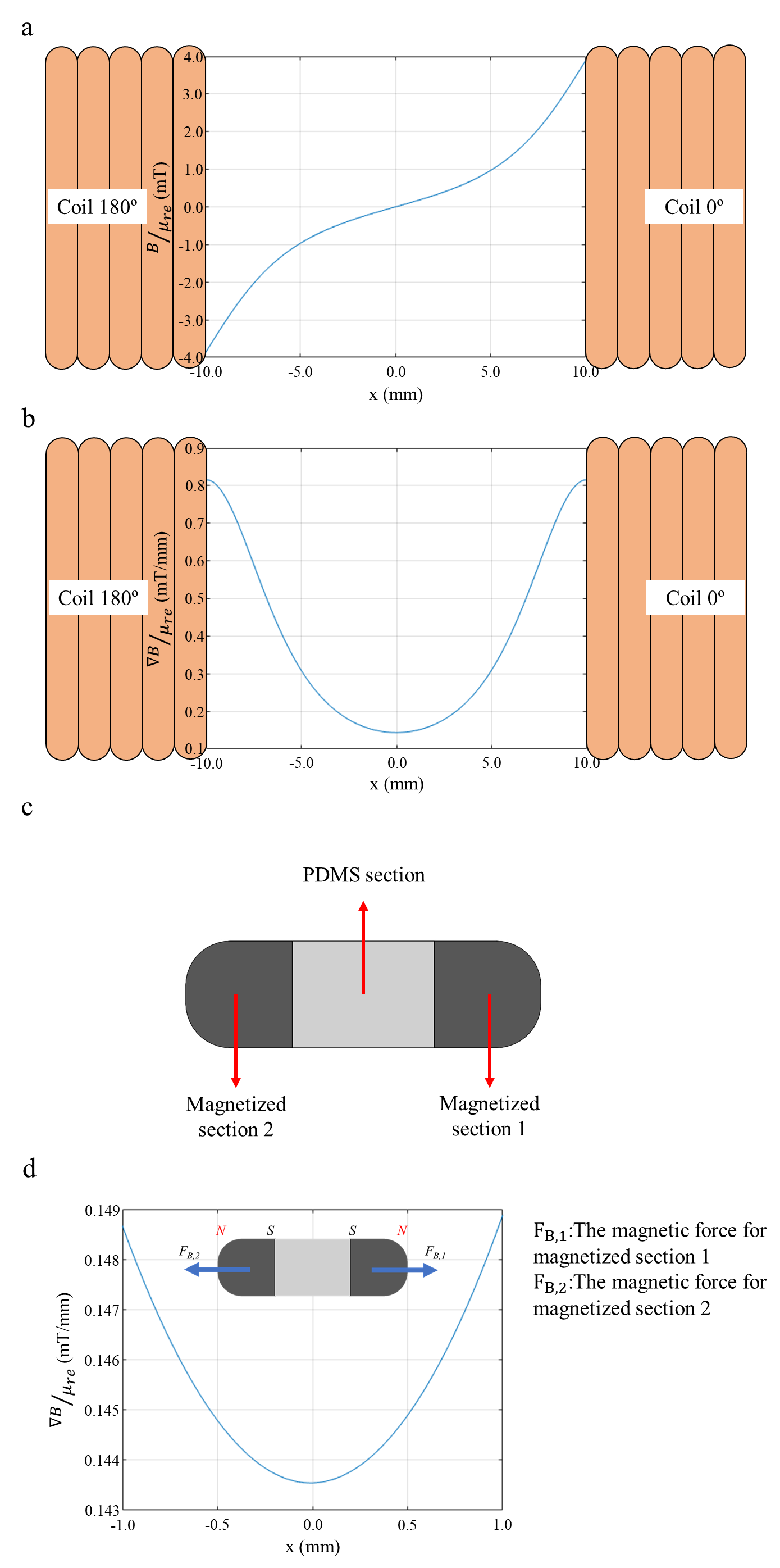


**Fig. S2** The magnetic field, magnetic field gradient distribution, and illustration of balance mechanism when the same current applied on coil 0˚ and coil 180˚. (a) The distribution of magnetic field per unit relative permeability (∇*B*/*μ_re_*)and (b) the distribution of magnetic field gradient per unit relative permeability (∇*B*/*μ_re_*) along the x-axis where the relative permeability *μ_re_* is defined as the ratio of the permeability of a specific medium to the permeability of free space. (c) Compositions of three sections in the microrobot. (d) Schematic illustration of the forces acting on the microrobot.

**1.2.2 Translational Motions**

To translate the microrobot along the positive x-direction, the magnetic intensity of the coil at 0˚ was modulated. The microrobot was placed at the midpoint between these two coils with the long axis parallel to the external magnetic field, as shown in Fig. S1(b). The current passed through the coil at 0˚ was intensified 1.25 times larger than the rest of the coils as shown in Fig. S3(a). Also, Fig. S3. shows that how the microrobot moved translationally towards the designated positions. The corresponding magnetic field and the magnetic field gradient are plotted in Fig. S4. The associated distribution of the magnetic field gradient is plotted in Fig. S4(b) where the gradient distribution was observed to be asymmetric. The magnitude of the magnetic force acting on the microrobot was determined using equation (2) in Section 1.2. It was found that the force acting on the magnetized section 1, F_B,1_ is larger than that acting on the magnetized section 2, F_B,2_, as shown in Fig. S4(c). As a result, the net magnetic force pointed rightwards, and the microrobot translated along the positive x-direction.


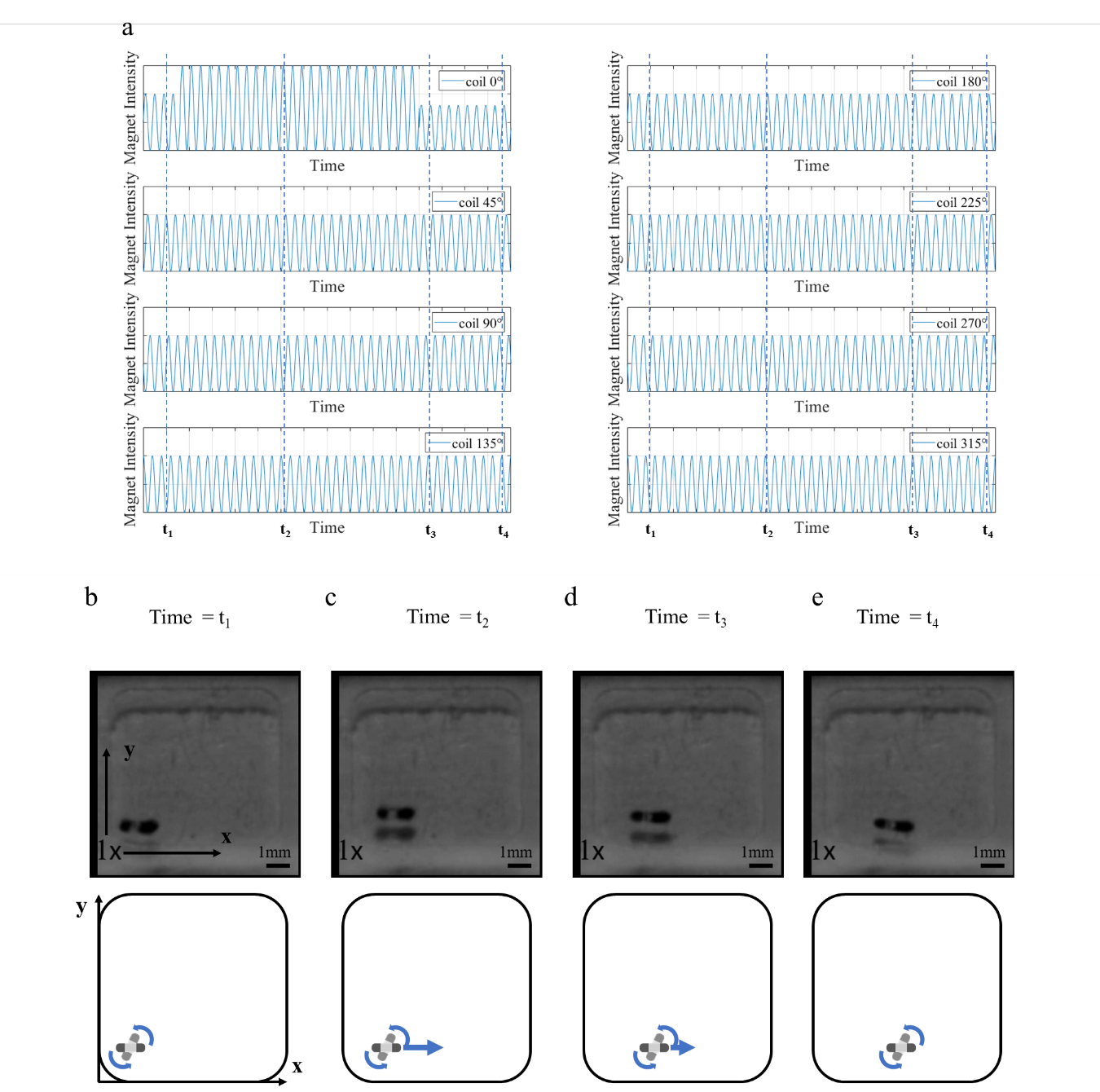


**Fig. S3** Demonstration of the microrobot translational motions. (a) The intensity waveform of the eight coils along with the highlighted time steps: t_1_, t_2_, t_3_, and t_4_. (b) When time = t_1_, the microrobot stayed in the origin. (c) At time = t_2_, the intensity of coil 0˚ increased, and the microrobot began to move to the right (along the positive x-direction) (d) At time = t_3_, the decreasing of the intensity of coil 0˚ slowed down the microrobot (e) At time = t_4_, the microrobot was held at the designated position.


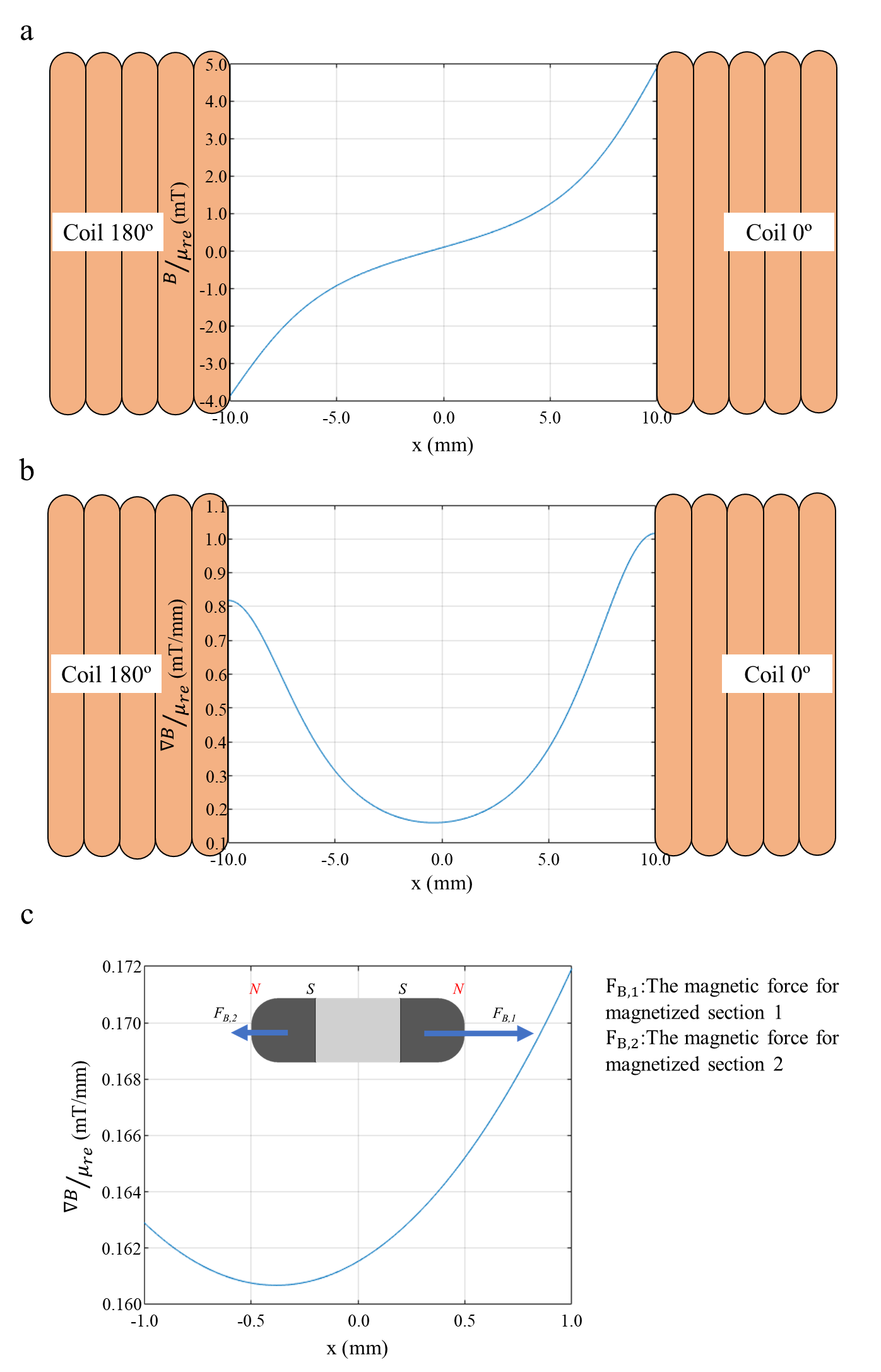


**Fig. S4** Calculaiton of the magnetic field, magnetic field gradient distributoin, and illustration of translation mechanism when the current through the coil 0˚ was 1.25 times stronger than the rest of the coils. (a) The distribution of magnetic field per unit relative permeability (∇*B*/*μ_re_*) and (b) the distribution of magnetic field gradient per unit relative permeability (∇*B*/*μ_re_*) along the x-axis, and (c) the schematic illustration of the forces acted on the microrobot.

**1.2.3 Rotational mechanism**

A rotational motion of the microrobot was achieved by altering the magnetic intensity of the coils sequentially the microrobot. Figure.S5 depicts the transformation of the microrobot from a horizontal position to a vertical position by counterclockwise rotation. To demonstrate the rotational motion of the microrobot, the coils at 0- and 180-degree were energized while the other coils were kept without no passing current. The magnitude of the current applied was identical for both active coils. The moments developed on the two magnetized sections of the microrobot was with same magnitude but in the opposite directions. When the initial state of the microrobot was not parallel to the x-axis, a net moment resulted in the rotational motion of the microrobot.


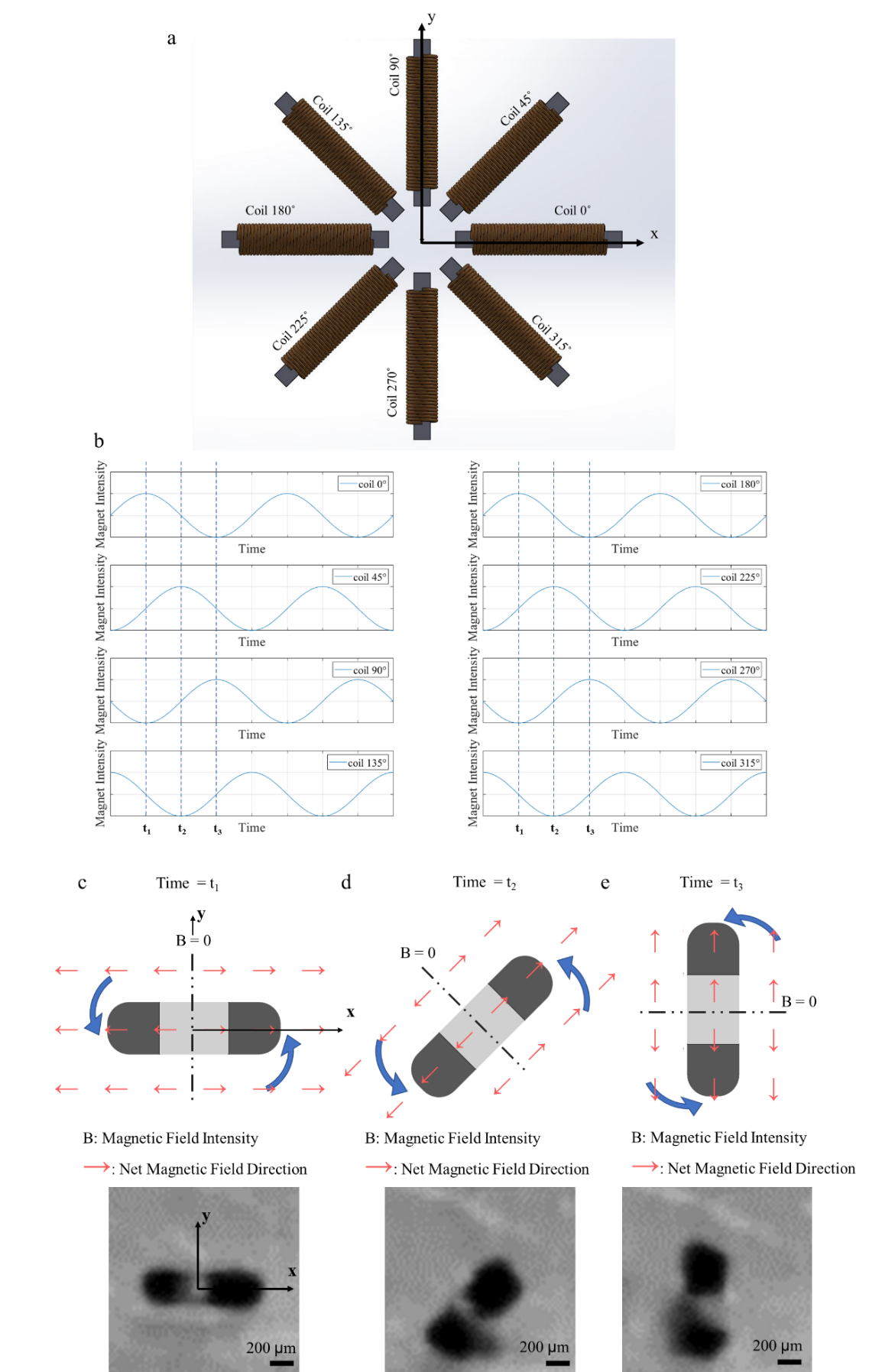


**Fig. S5** Demonstration of the rotational motions of the microrobot. (a) The configuration of the electromagnet system. (b) The intensity waveform of the eight coils along with the highlighted time steps: t_1_, t_2_, and t_3_. (c) When time = t_1_, the microrobot is parallel to the horizontal magnetic field direction. (d) At time = t_2_, the microrobot rotates according to the magnetic field. (e) When time = t_3_, the microrobot is parallel to the vertical magnetic field direction.

**1.2.4 Dynamic motion of the microrobot in inclined direction**

The combined translational and rotational motion of the microrobot at the inclined direction was achieved, and to demonstrate the dynamic motion of the microrobot in the inclined direction, two stages were used. Stage 1: Reorientation of the microrobot from the initial configuration to the desired angle of navigation. Stage 2: Dynamic motion of the microrobot in the desired angle of navigation. Figure.S6 depicts the different stages of the microrobot that undergoes a dynamic motion in an inclined direction. 45˚ angle-inclined navigation was considered for the demonstration purpose. The current of the same magnitude was applied to the coils placed at 45˚ and 225˚, and only this pair of coils were energized and the other coils were kept at the dead state. In consequence, the configuration of the microrobot was changed from the planar (horizontal) orientation to an inclined orientation of angle 45˚. The moments developed on the two magnetized sections of the microrobot were of the same magnitude but acted in the opposite directions. Thus, the equilibrium state of the microrobot was achieved at 45˚. To propel the microrobot in the forward direction, the current passed through the coil at 45˚ was intensified than the coil at 225˚. Thus, the dynamic motion of the microrobot was achieved at an angle of 45˚. Likewise, the dynamic motion can be achieved in all nine angles each with additional 45˚angle increment (0, 45, 90, 135, 180, 225, 270, 315, and 360).


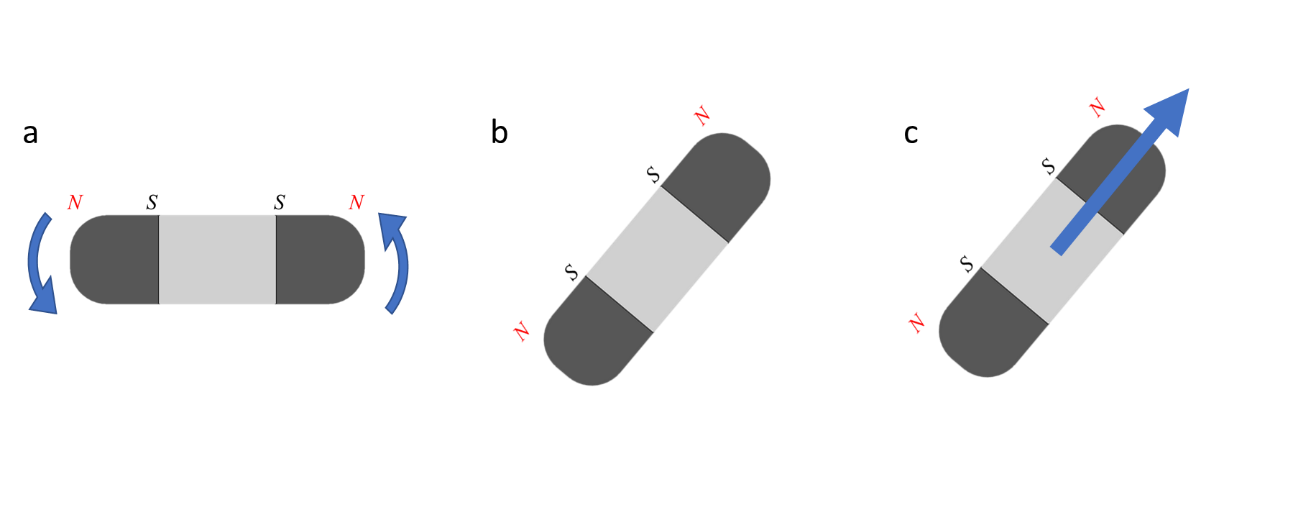


**Fig. S6** Stages of inclined navigation (a) initial configuration of the microrobot, (b) reorient microrobot at 45˚, and (c) dynamic motion of the microrobot at 45˚

**1.2.5 Discussion on the three tested waveforms**

**Table S1** Comparison of three different waveforms

| **No.** | **Modes/Waveforms** | **Waveform characteristics** | **Intensity shift** | **Comments on the stability of microrobot** |
| --- | --- | --- | --- | --- |
| 1 | Sawtooth | Linearly increased as a triangular wave and suddenly dropped as a square wave | Acute | The microrobot reached the target position in a short period, but less stable. |
| 2 | Sinusoidal | Smooth transition | Smooth | The microrobot exhibited smooth rotation and translation, and hence it is more stable. |
| 3 | Triangle | Linearly increased and dropped | Moderate | It is less stable than the sinusoidal waveform but more stable than the sawtooth waveform |

**2. Dynamics of the microrobot based on theoretical analysis**

A theoretical study on understanding the dynamics of the microrobot is discussed along with the comparison between the analytical solutions and the experiment results.

**2.1 Theoretical analysis:**

To analyze the microrobot dynamics analytically, a relationship between any of the dynamic's variables to the magnetic field intensity is introduced. This was done by equating the torque induced on the microrobot due to the magnetic force with magnetic flux density.

| $\tau=m\times B$ | (1) |
| --- | --- |

where $m$ is a vector that indicates the magnetic dipole pitch points from S (south pole) inside the microrobot to N (north pole) and $B$ is the magnetic flux density directed by the external magnetic field on the microrobot.

| $\tau=VM\times B$ | (2) |
| --- | --- |

Also, $m=VM$ which gives the following:

where $M$ is the magnetization of the object, and $V$ is the volume of the magnetized object (Field et al., 2019). Further, from the definition of outer product, obtain

| $\tau=V\left\vert M \right\vert\left\vert B \right\vert\sin\theta$ | (3) |
| --- | --- |

Now, to determine an experimental torque value, angular acceleration was employed. The dependency of torque with angular acceleration and this is done by equating the torque with moment of inertia. From the definition of torque, it is known:

| $\tau=Fr$ | (4) |
| --- | --- |

where *F* is the force and *r* is the radius in the case of rotation. From the Newton's second law of motion, it is known:

| 𝐹=𝑚$a_{t}$ | (5) |
| --- | --- |

The process of deriving the angular acceleration from instantaneous acceleration is described as the following.

| $a_{t}=\lim_{\Delta t\to0} \frac{\Delta V}{\Delta t}=\lim_{\Delta t\to0} \frac{r\Delta\omega}{\Delta t}=r\lim_{\Delta t\to0} \frac{\Delta\omega}{\Delta t}=ra$ | (6) |
| --- | --- |

By substituting equation (6) and (5) into equation (4), we obtain

| $\tau=mar^{2}$ | (7) |
| --- | --- |

In the classical mechanics, the kinetic energy of a point mass or rigid body can be expressed as

| $E=\frac{1}{2}mV^{2}$ | (8) |
| --- | --- |

For a rotating object, the kinetic energy is achieved by replacing $V$ in equation (7) with a radius $r$ multiplied with the angular velocity$\omega$.

| $E=\frac{1}{2}mr^{2}\omega^{2}$ | (9) |
| --- | --- |

The rotational kinetic energy can be expressed as

| $E_{rotation}=\frac{1}{2}I\omega^{2}$ | (10) |
| --- | --- |

By comparing equation (9) and (10), obtain:

| $I=mr^{2}$ | (11) |
| --- | --- |

where *I* is the moment of inertia around the axis of rotation. By equating equation (11) with equation (7), we obtain

| $\tau=Ia$ | (12) |
| --- | --- |

Angular acceleration a can be expressed as

| $a=\frac{\omega}{\Delta t}=\frac{\Delta\theta}{{\Delta t}^{2}}$ | (13) |
| --- | --- |

where $\Delta\theta$ – rotating angle of the microrobot from its initial position

$\Delta t$ – time taken from $\Delta\theta$ rotation (which is obtain from the experiment)

Thus, by substituting equation (13) in equation (12), we obtain

| $\tau=I\frac{\Delta\theta}{{\Delta t}^{2}}$ | (14) |
| --- | --- |

By using equation (14) values for the experimental torque can be obtained.

**2.2 Comparison between the theoretical solutions with the experimental results**

Two microrobots were prepared in this test. **Microrobot 0**: The microrobot consists of three segments where the two ends of the microrobot were filled with the mixture of the magnetic particles and PDMS while the middle section was filled with PDMS only. This design has the same features as demonstrated in the major text. **Microrobot 1**: The mixture of the magnetic particles and PDMS was only on one side and the rest sections of the microrobot was pure PDMS. Table. S2 depicts the comparison results between these two types from the experimental and analytical perspectives. Results show that the experimental result is consistent with the theoretical results as for the microrobot 0 test the difference is only 0.595% and it is 4.57% in the microrobot 1 test.

**Table S2** Comparison of theoretical and experiment results for two types of microrobot tests.

| **Microrobot 0** | | | | **Microrobot 1** | | | |
| --- | --- | --- | --- | --- | --- | --- | --- |
| **Angle of rotation**  $\boldsymbol{\theta}$ | **Ratio of theoretical torque**  $\boldsymbol{\tau}_{\boldsymbol{th}_{\boldsymbol{ratio}}}$ | **Ratio of experimental torque**  $\boldsymbol{\tau}_{\boldsymbol{ex}_{\boldsymbol{ratio}}}$ | **Relative percentage difference**  **RPD (%)** | **Angle of rotation**  $\boldsymbol{\theta}$ | **Ratio of theoretical torque**  $\boldsymbol{\tau}_{\boldsymbol{th}_{\boldsymbol{ratio}}}$ | **Ratio of experimental torque**  $\boldsymbol{\tau}_{\boldsymbol{ex}_{\boldsymbol{ratio}}}$ | **Relative percentage difference**  **RPD (%)** |
| $\theta_{1}$=$24.3^{\circ}$ | 0.682 | 0.678 | 0.595 | $\theta_{1}$=$53.8^{\circ}$ | 0.922 | 0.881 | 4.57 |
| $\theta_{2}$=$142.9^{\circ}$ |  |  |  | $\theta_{2}$=61.1$^{\circ}$ |  |  |  |

**2.2.1 Calculation**

Theoretical torque

We considered two angles of rotation $\theta_{1}$=$24.3^{\circ}$ and $\theta_{2}$=$142.9^{\circ}$

| $\frac{\tau_{th(24.3^{\circ})}}{\tau_{th(142.9^{\circ})}}=\frac{VMB\sin24.3^{\circ}}{VMB\sin142.9^{\circ}}=\frac{\sin24.3^{\circ}}{\sin142.9^{\circ}}=0.6822$ |  |
| --- | --- |

Experimental torque

For the same values of $\theta$ as considered above,

| $\frac{\tau_{ex(24.3^{\circ})}}{\tau_{ex(142.9^{\circ})}}=\frac{Ia_{1}}{Ia_{2}}=\frac{\frac{\omega_{1}}{\Delta t_{1}}}{\frac{\omega_{2}}{{\Delta t}_{2}}}=\frac{\frac{\Delta\theta_{1}}{{{\Delta t}_{1}}^{2}}}{\frac{\Delta\theta_{2}}{{{\Delta t}_{2}}^{2}}}=\frac{\frac{24.3}{{0.0334}^{2}}}{\frac{142.9}{{0.0667}^{2}}}=0.678$ |  |
| --- | --- |
| $RPD \left( \% \right)=\left( \frac{\left\vert\boldsymbol{\tau}_{\boldsymbol{th}_{\boldsymbol{ratio}}}\boldsymbol{-}\boldsymbol{\tau}_{\boldsymbol{ex}_{\boldsymbol{ratio}}} \right\vert}{\frac{\left( \boldsymbol{\tau}_{\boldsymbol{th}_{\boldsymbol{ratio}}}\boldsymbol{+}\boldsymbol{\tau}_{\boldsymbol{ex}_{\boldsymbol{ratio}}} \right)}{2}} \right)*100=0.595$ |  |

| (a) | 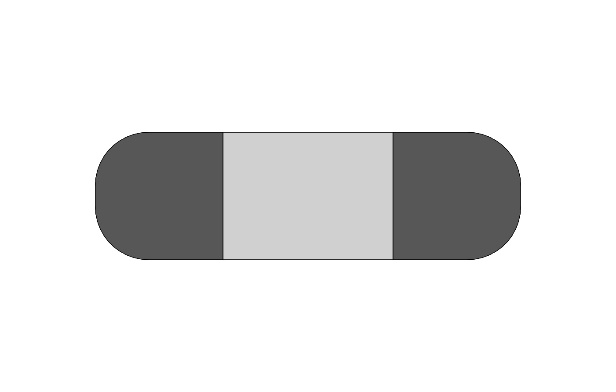 | (b) | 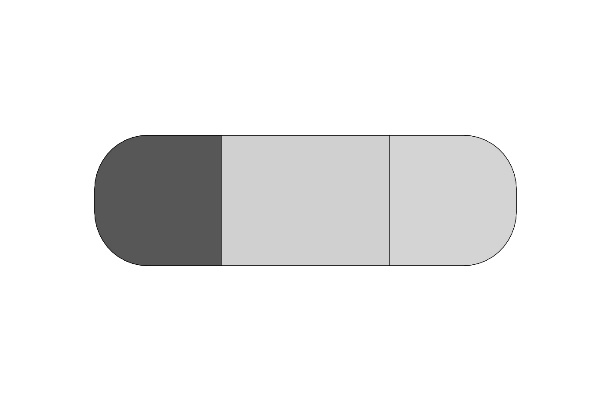 |
| --- | --- | --- | --- |
| **Fig. S7** Designs of two microrobots. (a) Microrobot 0: with two magnetic sections on both side and one PDMS section in the middle (presented in the majortext). (b) Microrobot 1: With a magnetic section only in one section. Others are PDMS. | | | |


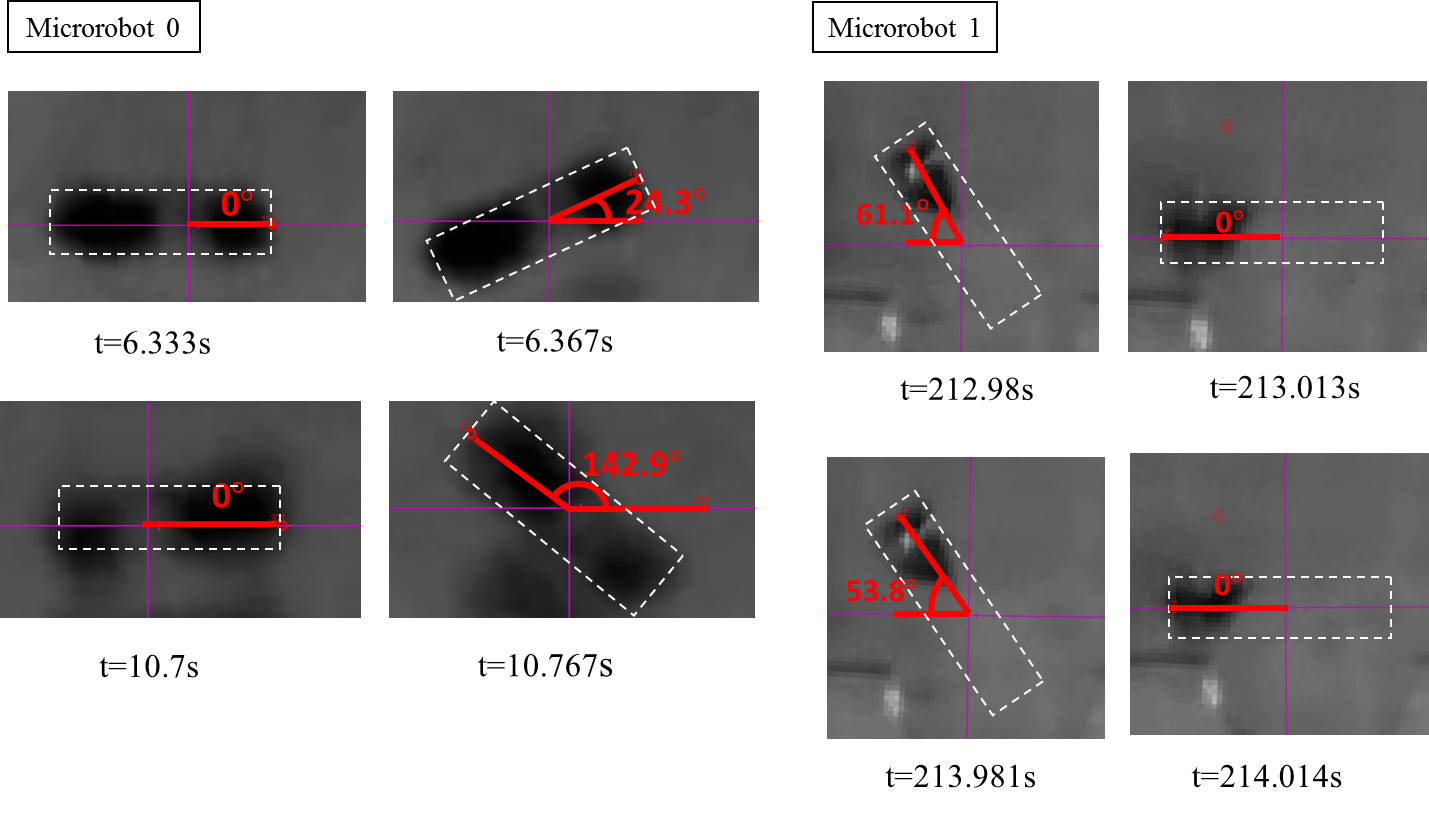


**Fig. S8** Images of the microrobot with tested angles of rotation
